# Supplementary figures and images for: The p150 Isoform of ADAR1 Blocks Sustained RLR signaling and Apoptosis during Influenza Virus Infection
Source: PLoS Pathog. 2020 Sep 8;16(9):e1008842. doi: 10.1371/journal.ppat.1008842 (PMC7500621; doi:10.1371/journal.ppat.1008842)

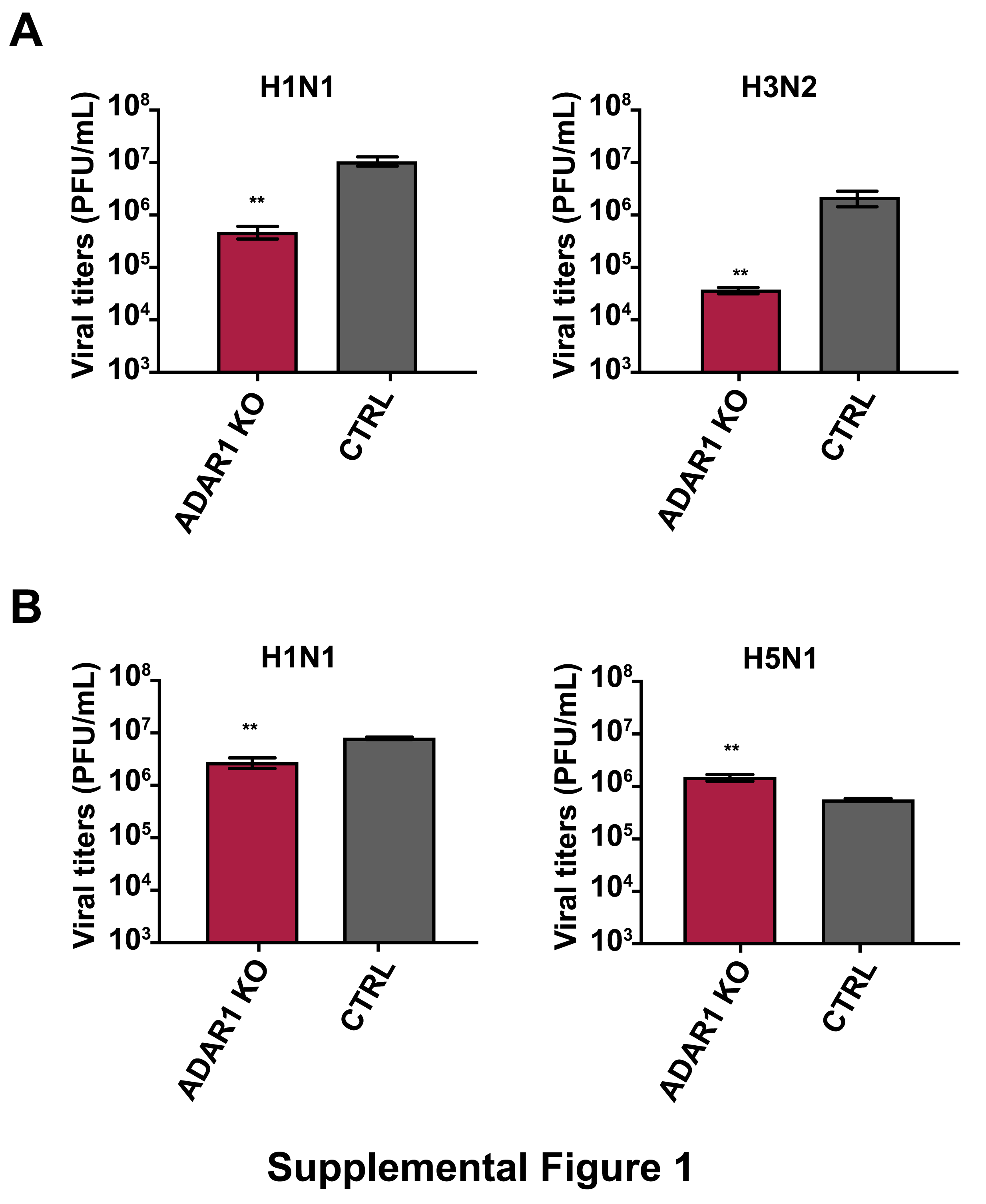

Supplement: S1 Fig — (A) ADAR1 KOs and CTRL A549s were infected with H1N1 (MOI = 0.01) and H3N2 (MOI = 0.01). Viral titers were measured at 48 hours. (B) Assessment of single-cycle viral replication. ADAR1 KOs and CTRL A549s were infected with H1N1 (MOI = 1) or H5N1 (MOI = 1) and maintained in infection media without TPCK-trypsin. At 24h, supernatants were collected and treated with TPCK-trypsin prior to measurement of viral titers. Data are represented as mean titer of triplicate samples ± SD. * denotes p-value ≤ 0.5. ** denotes p-value ≤ 0.01. *** denotes p-value ≤ 0.001. NS denotes p-value ≥ 0.05. Data are representative of at least three independent experiments. (TIF) [file ppat.1008842.s001.tif]

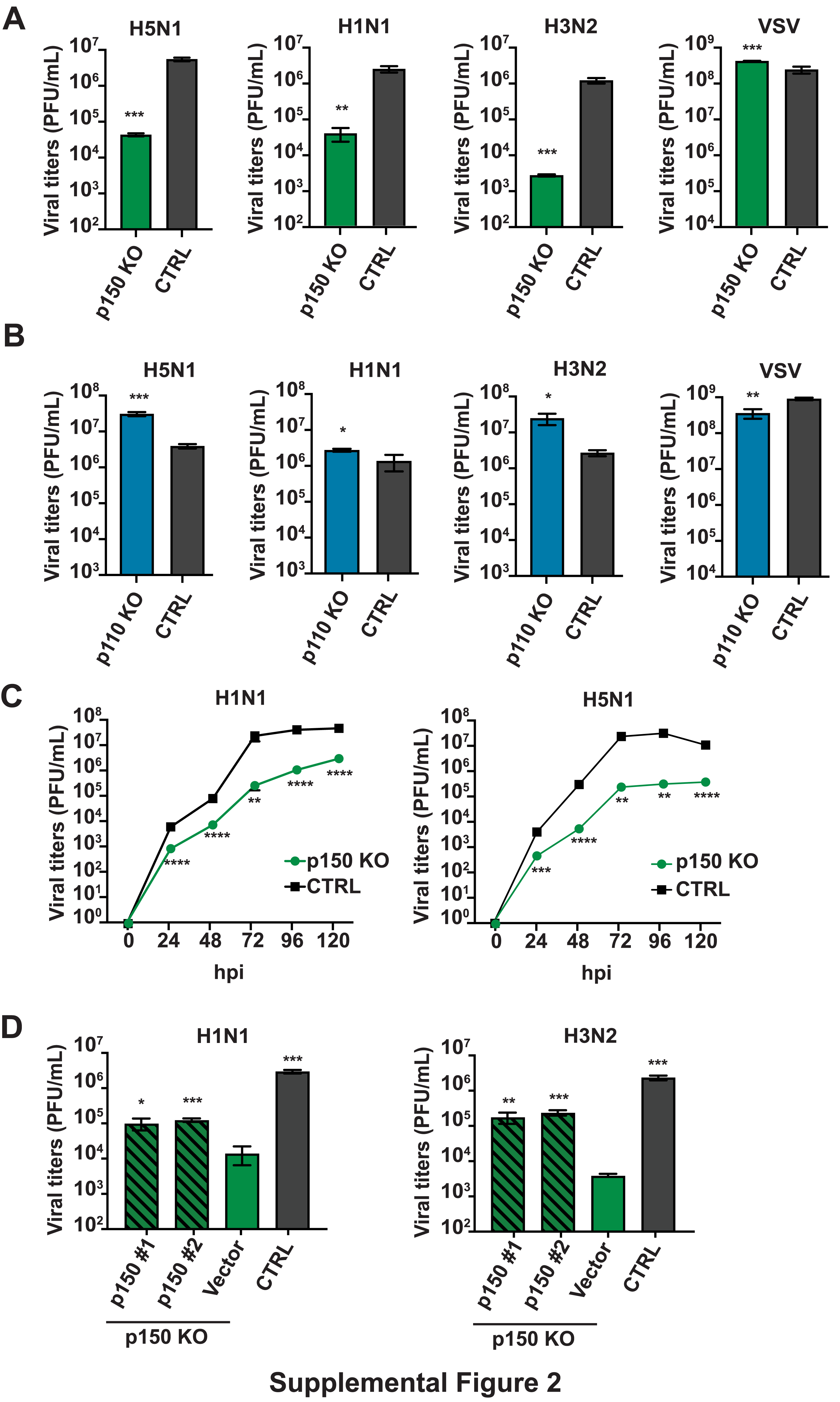

Supplement: S2 Fig — (A) Assessment of viral replication in p150 KO and CTRL A549s. p150 KO and CTRL A549s were infected with H5N1 (MOI = 0.001), H1N1 (MOI = 0.01), H3N2 (MOI = 0.01), and VSV (MOI 0.001) and viral titers were measured at 48 hpi. (B) Assessment of viral replication in p110 KO and CTRL A549s. p110 KO and CTRL A549s were infected with H5N1 (MOI = 0.001), H1N1 (MOI = 0.01), H3N2 (MOI = 0.01), and VSV (MOI 0.001) and viral titers were measured at 48 hpi. (C) p150 KOs and CTRL A549s were infected with H1N1 (MOI = 0.01) and H5N1 (MOI = 0.001) and viral titers were measured at the indicated times post infection. (D) Two clones of p150 KOs complemented with wildtype p150, empty vector p150 KOs, and CTRL A549s were infected with H1N1 (MOI = 0.01) and H3N2 (MOI = 0.01) and viral titers were measured at 48 hpi. Data are represented as mean titer of triplicate samples ± SD. * denotes p-value ≤ 0.5. ** denotes p-value ≤ 0.01. *** denotes p-value ≤ 0.001. NS denotes p-value ≥ 0.05. Data are representative of at least three independent experiments. (TIF) [file ppat.1008842.s002.tif]

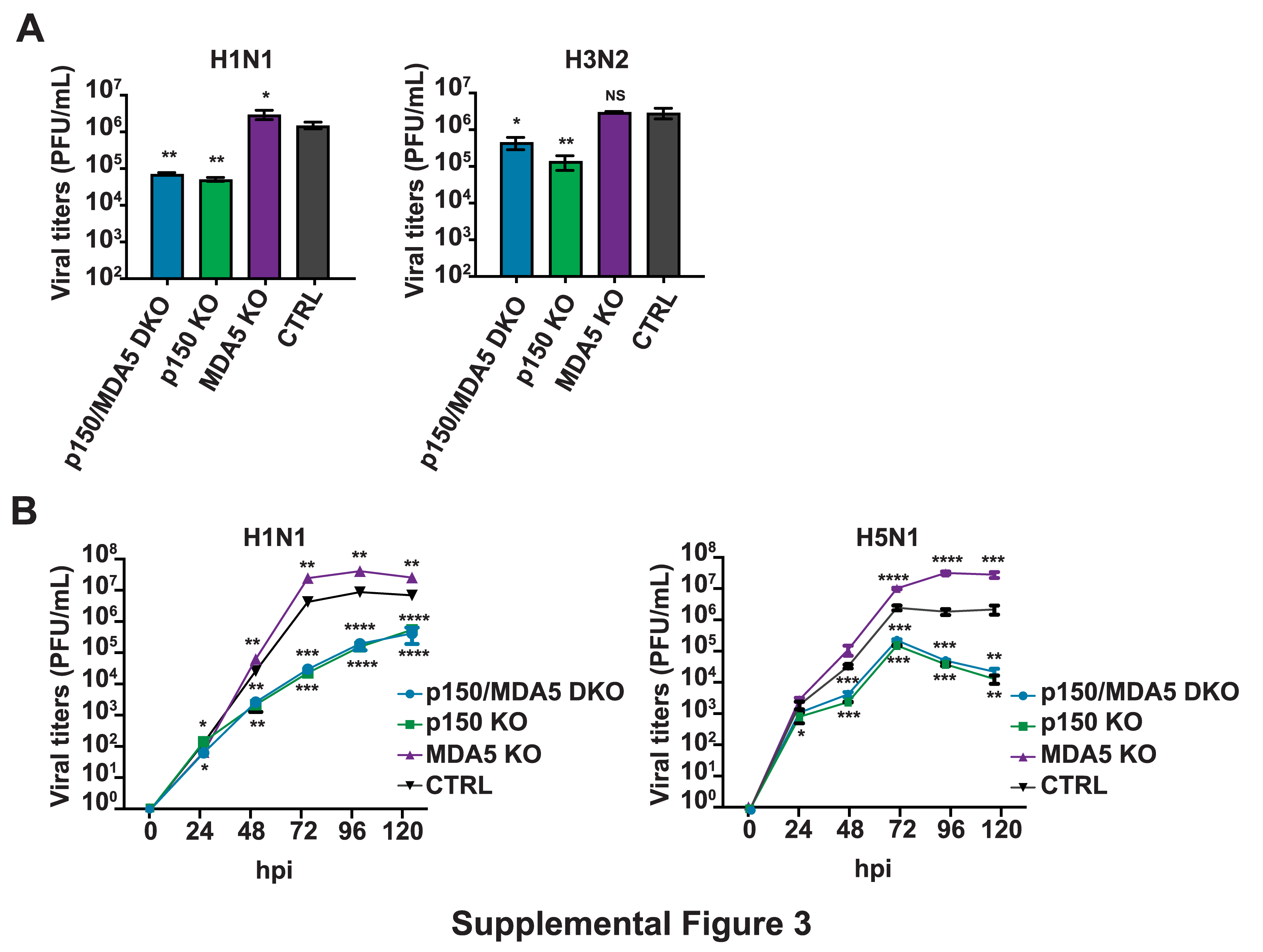

Supplement: S3 Fig — (A) Assessment of viral replication in various KOs. p150/MDA5 DKOs, p150 KOs, MDA5 KOs, and CTRL A549s were infected with H1N1 (MOI = 0.01) and H3N2 (MOI = 0.01). Viral titers were measured at 48 hours. (B) Viral replication kinetics in various KOs. p150/MDA5 DKOs, p150 KOs, MDA5 KOs, and CTRL A549s were infected with H1N1 (MOI = 0.01) and H3N2 (MOI = 0.01). Viral titers were measured at the indicated time points post infection. Data are represented as mean titer of triplicate samples ± SD. * denotes p-value ≤ 0.5. ** denotes p-value ≤ 0.01. *** denotes p-value ≤ 0.001. NS denotes p-value ≥ 0.05. Data are representative of at least three independent experiments. (TIF) [file ppat.1008842.s003.tif]

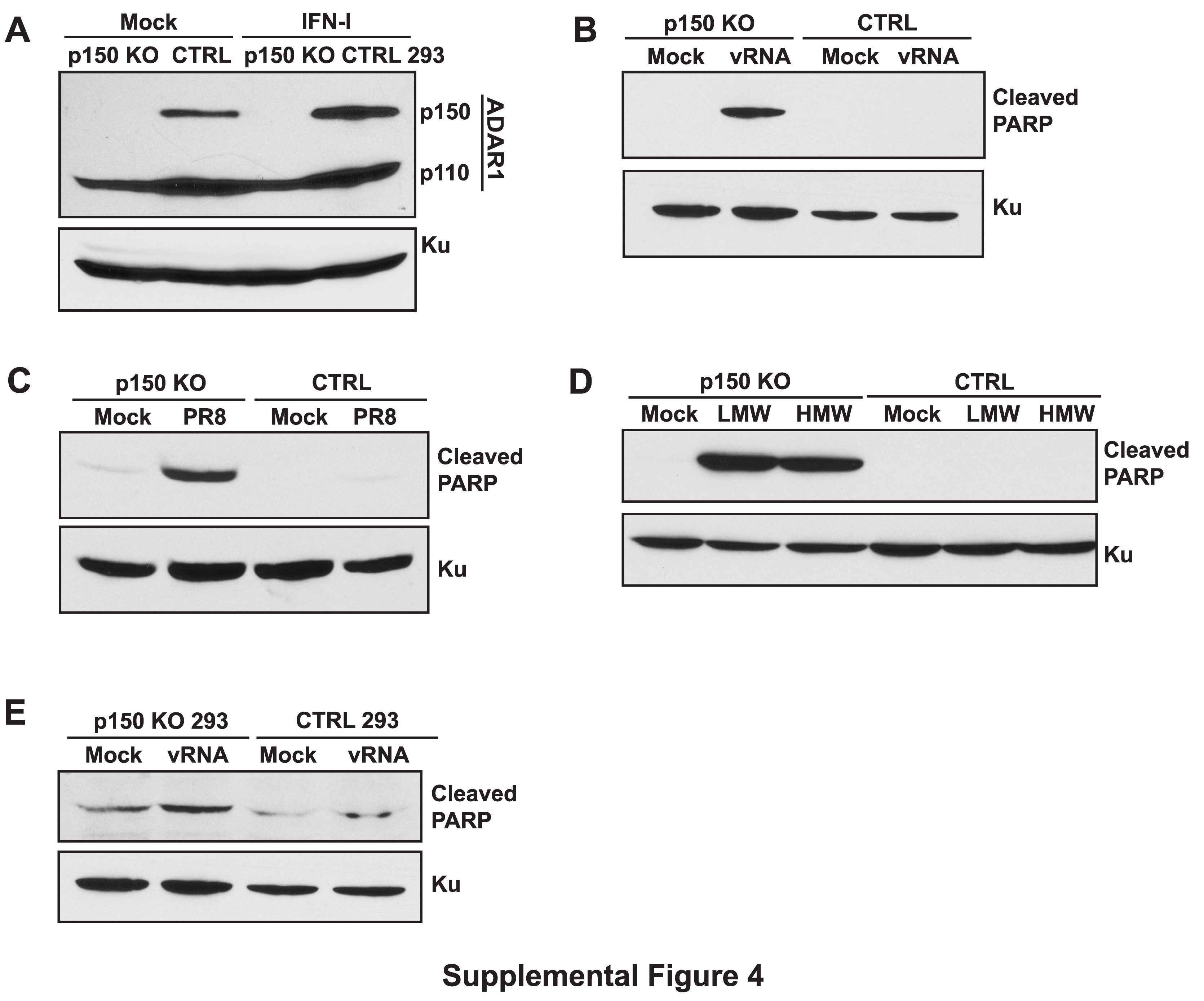

Supplement: S4 Fig — (A) Western blot analysis of ADAR1 expression in p150 KOs and CTRL 293s. p150 KOs, and CTRL 293s were mock treated or treated with IFN for 24 hours and expression of ADAR1 was examined by western blot. Expression of Ku is shown as a loading control. (B-D) Western blot analysis of PARP cleavage upon RLR stimulation. (B) PARP cleavage in p150 KOs following IAV vRNA transfection. p150 KOs and CTRL A549s were transfected with H1N1 vRNA. Lysates were collected at 24 hours post transfection. (C) PARP cleavage p150 KOs following H1N1 infection. p150 KOs and CTRL A549s were infected with H1N1 (MOI = 1). Lysates were collected at 40 hours post infection. (D) PARP cleavage in p150 KO following poly I:C transfection. p150 KOs and CTRL A549s were transfected with LMW or HMW pI:C. Lysates were collected at 24 hours post transfection. (E) PARP cleavage in p150 KO 293s following IAV vRNA transfection. p150 KOs and CTRL 293s were transfected with H1N1 vRNA. Lysates were collected at 24 hours post transfection. (TIF) [file ppat.1008842.s004.tif]
